# Dual Role of Endogenous Serotonin in 2,4,6-Trinitrobenzene Sulfonic Acid-Induced Colitis

**DOI:** 10.3389/fphar.2016.00068

**Published:** 2016-03-22

**Authors:** Alberto Rapalli, Simona Bertoni, Valentina Arcaro, Francesca Saccani, Andrea Grandi, Valentina Vivo, Anna M. Cantoni, Elisabetta Barocelli

**Affiliations:** ^1^Dipartimento di Farmacia, Università degli Studi di ParmaParma, Italy; ^2^Dipartimento di Scienze Medico-Veterinarie, Università degli Studi di ParmaParma, Italy

**Keywords:** intestine, inflammation, 5-HT_2A_ receptor, 5-HT_1A_ receptor, apoptosis

## Abstract

**Background and Aims:** Changes in gut serotonin (5-HT) content have been described in Inflammatory Bowel Disease (IBD) and in different experimental models of colitis: the critical role of this monoamine in the pathogenesis of chronic gastrointestinal inflammation is gradually emerging. Aim of the present study was to evaluate the contribution of endogenous 5-HT through the activation of its specific receptor subtypes to the local and systemic inflammatory responses in an experimental model of IBD.

**Materials and Methods:** Colitis was induced by intrarectal 2,4,6-TriNitroBenzene Sulfonic acid in mice subacutely treated with selective antagonists of 5-HT_1A_ (WAY100135), 5-HT_2A_ (Ketanserin), 5-HT_3_ (Ondansetron), 5-HT_4_ (GR125487), 5-HT_7_ (SB269970) receptors and with 5-HT_1A_ agonist 8-Hydroxy-2-(di-*n*-propylamino)tetralin.

**Results:** Blockade of 5-HT_1A_ receptors worsened TNBS-induced local and systemic neutrophil recruitment while 5-HT_1A_ agonist delayed and mitigated the severity of colitis, counteracting the increase in colonic 5-HT content. On the contrary, blockade of 5-HT_2A_ receptors improved global health conditions, reduced colonic morphological alterations, down-regulated neutrophil recruitment, inflammatory cytokines levels and colonic apoptosis. Antagonism of 5-HT_3_, 5-HT_4_, and 5-HT_7_ receptor sites did not remarkably affect the progression and outcome of the pathology or only slightly improved it.

**Conclusion:** The prevailing deleterious contribution given by endogenous 5-HT to inflammation in TNBS-induced colitis is seemingly mediated by 5-HT_2A_ and, to a lesser extent, by 5-HT_4_ receptors and coexists with the weak beneficial effect elicited by 5-HT_1A_ stimulation. These findings suggest how only a selective interference with 5-HT pro-inflammatory actions may represent an additional potential therapeutic option for intestinal inflammatory disorders.

## Introduction

Serotonin (or 5-Hydroxytryptamine, 5-HT) is a well-known mediator both in the brain and in the periphery. In the periphery, 5-HT plays a pivotal role as paracrine mediator and neurotransmitter; in particular, in the GI tract, where most of the monoamine resides, 5-HT is stored in EC cells and serotonergic neurons of the myenteric plexus and is released for the physiological regulation of gut motility, secretion and perception (De Ponti, 2004). Abnormalities in serotonergic signaling have been reported in a number of functional and inflammatory GI disorders, both clinical and experimental, like IBS (Spiller, 2008), mesenteric ischemia-reperfusion (Teramoto et al., 1998; Bertoni et al., 2007; Bertoni et al., 2014), celiac disease (Coleman et al., 2006) and IBD (Belai et al., 1997). IBD, including CD and UC, is a chronic inflammatory disorder with relapsing and remitting course, one of the most serious diseases affecting human bowel: its incidence is increasing worldwide and, up to now, remains incurable. Enhanced or, alternatively, lowered EC cell numbers and 5-HT content have been reported in association with both CD and UC (El-Salhy et al., 1997; Magro et al., 2002). In different experimental models of colitis increased 5-HT content has been observed (Oshima et al., 1999; Linden et al., 2003). Accordingly, mice lacking 5-HT re-uptake transporter (SERT) exhibited exacerbation of chemically and genetically induced colitis (Bischoff et al., 2009); conversely, a lower damage was reported in mice with reduced availability of gut 5-HT, through genetic deletion or pharmacological inhibition of Tph1 (Ghia et al., 2009; Margolis et al., 2014; Kim et al., 2015).

Decisive to its involvement in the patho-physiology of a chronic inflammatory condition like IBD, triggered by a dysregulated immune response (Abraham and Cho, 2009), is the influence of 5-HT both on GI and on immune system functions. Indeed, 5-HT exerts a wide range of effects in the gut through the interaction with multiple receptor subtypes present on smooth muscle, enteric neurons and enterocytes but also on innate and adaptive immune cells, like monocytes, macrophages, dendritic cells, B and T lymphocytes (Manocha and Khan, 2012; Arreola et al., 2015). Despite this wealth of information, the role played by the distinct 5-HT receptor subtypes in IBD pathogenesis and progression has yet to be unraveled.

Aim of the present work was therefore to evaluate the contribution given by endogenous 5-HT through the activation of specific receptor subtypes, shown to be expressed in the GI tract, to the local and systemic inflammatory responses induced in an acute murine model of CD. Accordingly, we first verified the increase in colonic 5-HT content in TNBS-induced colitis; second, we evaluated the effects produced by the repeated administration of selective antagonists of 5-HT_1A_ (WAY100135), 5-HT_2A_ (Ketanserin), 5-HT_3_ (Ondansetron), 5-HT_4_ (GR125487), and 5-HT_7_ (SB269970) receptors in the same experimental model. In order to gain further insight into the beneficial action shown by Ketanserin, we tested its ability to prevent colonic apoptosis, typically increased in clinical and experimental colitis and regarded as contributing factor in IBD pathogenesis (Günther et al., 2013), and to promote epithelial cell protection via the stress-inducible HSP70, whose expression has been reported to be down-regulated in IBD (Hu et al., 2007). Finally, given the massive leukocyte recruitment triggered by blockade of 5-HT_1A_ receptors in colitic animals, we investigated the potential protective activity afforded by their stimulation through the administration of the selective 5-HT_1A_ agonist 8-OH-DPAT.

## Materials and Methods

### Animals

Swiss CD1 female mice, 6–10 weeks old (Charles River Laboratories, Calco, Italy) weighing 25–30 g, were housed five per cage, under standard conditions (12:12 h light–dark cycle, water and food *ad libitum*, 22–24°C). All the experiments were performed in accordance with Guiding Principles in the Care and Use of Animals (DL116/92) and were authorized by the local Animal Care Committee “Organismo preposto al benessere degli animali” and by Italian Ministry of Health.

### Induction of Colitis and Experimental Protocol

To induce colitis, mice were lightly anesthetized with diethyl ether and a 10-cm long PE-50 tubing attached to a tuberculin syringe was inserted 2 cm into the colon; 50 μl of a 10% (w/v) TNBS solution in 50% ethanol was administered to mice, maintained in a vertical head-down position for 1 min to avoid leakage of the haptenating agent. Normal (N) animals, receiving 50 μl saline (0.9% NaCl) intrarectally, were included in the study as reference physiological group.

Mice were randomly assigned to the N group (16 animals), receiving subcutaneously saline (10 ml/kg), or to the following experimental groups of colitic animals: saline (C, 10 ml/kg; 16 animals), WAY100135 (W, 5 mg/kg; 12 animals), Ketanserin (K, 5 mg/kg; 12 animals), Ondansetron (O, 10 mg/kg; 12 animals), GR125487 (G, 10 mg/kg; 12 animals), SB269970 (S, 10 mg/kg; 12 animals) and 8-OH-DPAT (OH, 1 mg/kg; 12 animals). The drugs, subcutaneously administered at doses chosen according to our preliminary experiments (data not shown) as effective and not toxic, were repeated twice daily, starting 1 h after TNBS instillation. Animals were daily weighed and wellness status was assessed by unaware observers. Mice were euthanized 3 days after TNBS administration by CO_2_ inhalation, and organs (colon, liver, lungs) and blood samples were collected for macroscopic, microscopic, and biochemical analyses. In particular, for colon assays, each group of animals was randomly subdivided in three subgroups: colons excised from each subset was reserved either for histological analysis, for MPO activity determination or for cytokines assays. In N, C, W, K, and OH subsets randomly assigned to cytokines determination, colons were longitudinally sectioned in two halves, each one being reserved for cytokines determination or, respectively, for 5-HT and SP levels quantification (N, C, W, and OH subgroups) or for Caspase-3 and HSP70 assays (N, C and K subgroups).

### Evaluation of Inflammation

#### Disease Activity Index

Disease activity index is an evaluation of the pathology severity, based on the daily assignment of a score according to Cooper’s modified method (Cooper et al., 1993), on the basis of body weight loss, stool consistency, and rectal bleeding. The scores were quantified as follows: stool consistency: 0 (normal), 1 (soft), 2 (liquid); body weight loss: 0 (<5%), 1 (5–10%), 2 (10–15%), 3 (15–20%), 4 (20–25%), 5 (>25%) and rectal bleeding: 0 (absent), 1 (present). Mice were scored blindly by two investigators.

#### Macroscopic Score (MS)

After the euthanasia, the colon was removed and opened along the mesenteric line, gently flushed with saline solution and immediately evaluated for the presence of colonic inflammation. MS was quantified by a blinded investigator, according to previously published criteria (Wallace et al., 1989; Khan et al., 2002), as the sum of scores (max = 12) attributed to the presence of points of stenosis and hypertrophic zones (0, absent; 1, 1 stenosis; 2, 2 stenoses; 3, more than 2 stenoses), mucus (0, absent; 1, present), adhesions (0, absent; 1, 1 adhesion between colon and other intra-abdominal organs; 2, 2 adhesions; 3, more than 2 adhesions), intraluminal hemorrhage (0, absent; 1, present), erythema (0, absent; 1, area < 1 cm^2^; 2, area > 1 cm^2^), ulceration and necrosis zones (0, absent; 1, area < 1 cm^2^; 2, area > 1 cm^2^).

#### Colon Length and Thickness

To evaluate muscular contraction and deposition of amorphous material induced by massive inflammation, the length of colon, and cecum and their weight were measured, while weight/length ratio was calculated to estimate colon thickness (Bischoff et al., 2009).

#### Colonic and Hepatic Edema

Colon and liver edemas were measured according to Moore–Olufemi modified method (Moore-Olufemi et al., 2005). After euthanasia, organs were rinsed with saline solution, gently milked dry, and 1 cm long colon segments and a portion of liver were cut and weighed immediately (wet weight, WW). Tissues were then allowed to dry for 72 h at room temperature. Dry weights (DW) were measured and used to determine tissue fluid content from the following wet to dry ratio: (WW–DW)/DW.

#### Colonic and Pulmonary MPO Activity Assay

Myeloperoxidase activity, an indicator of tissue neutrophil accumulation, was determined according to Krawisz’s modified method (Krawisz et al., 1984). After being weighed, each colonic and lung sample was homogenized in ice-cold potassium phosphate buffer (100 mM, pH 7.4) containing aprotinin 1 μg/ml (1:10, v/v) and centrifuged for 20 min at 12500 RCF at 4°C. Pellets were re-homogenized in five volumes of ice-cold potassium phosphate buffer (50 mM, pH 6) containing 0.5% hexadecylthrimethyl-ammoniumbromide and aprotinin 1 μg ml^-1^. The samples were subjected to three cycles of freezing and thawing, and then centrifuged for 30 min at 15500 RCF at 4°C. 100 microliter of the supernatant was then allowed to react with 900 μl of a buffer solution containing *o*-dianisidine (0.167 mg ml^-1^) and 0.0005% H_2_O_2_. Each assay was performed in duplicate and the rate of change in absorbance was measured spectrophotometrically at 470 nm (Jenway, mod. 6300, Dunmow, Essex, England). The sensitivity of the assay was 10 mU/ml, 1 unit of MPO being defined as the quantity of enzyme degrading 1 μmol of peroxide per minute at 25°C. Data were normalized with edema values and expressed as U/g of dry weight tissue.

#### Colonic Cytokines Levels

After euthanasia, colon segments were homogenized for 1 min in 700 μl of tissue lysis buffer containing 20 mM Tris, 150 mM NaCl, 1% Nonidet P-40, 0.5% sodium deoxycholate, 1 mM EDTA and 0.1% SDS (pH 7.5) and protease inhibitors cocktail (pepstatin, aprotinin, and leupeptin 1 μg ml^-1^). Samples were then centrifuged for 20 min at 16500 RCF at 4°C and the supernatant was collected. Total protein concentration was quantified using Pierce BCA protein assay kit (ThermoFisher Scientific Inc., MA, USA) and each sample diluted to 5 mg/ml. IL-1β, TNFα, IFNγ, and IL-10 colonic concentrations were then determined in duplicate in 100 μl samples, using commercially available ELISA kits (PRODOTTI GIANNI S.p.A., Milano, Italy), according to the manufacturer’s protocol, with assays sensitivity, respectively, of 1 pg/ml (IL-1β), 8 pg/ml (TNFα), 10 pg/ml (IFNγ), and 30 pg/ml (IL-10). Results were expressed as pg/mg protein.

#### Plasmatic Cytokines Levels

Blood was collected by cardiac puncture after CO_2_ euthanasia, using heparin as anticoagulant (150 IU/ml, 1:9 v/v), and centrifuged at 6500 RCF for 15 min to obtain plasma. Plasma samples were stored at –20°C until the day of the assay. IL-1β and TNFα plasmatic concentrations were determined in duplicate using commercially available ELISA kits according to the procedures supplied by the manufacturer (PRODOTTI GIANNI S.p.A., Milano, Italy) and the results expressed as pg/ml of plasma.

#### Colonic 5-HT Levels

5-HT concentration was determined in the supernatant of colon homogenates with a commercially available EIA kit (Abnova) according to the manufacturer’s protocol. Samples were weighed, homogenized for 1 min in 1 ml of ice-cold 0.01 M HCl solution containing 1% ascorbic acid and centrifuged at 4000 RCF at 4°C for 15 min. Enzymatic reaction products were determined in duplicate spectrophotometrically at 450 nm, the assay sensitivity being 5 ng/ml (Jenway, mod. 6300, Dunmow, Essex, England). Results were normalized with colonic edema values and expressed as μ/g dry weight tissue.

#### Colonic SP Levels

Substance P concentration was determined in the supernatant of colon homogenates with a commercially available EIA kit (Cayman Chemicals) following manufacturer’s protocol. Samples were weighed, homogenized for 1 min in 1 ml of ice-cold PBS (pH 7.4) solution containing protease inhibitors cocktail (pepstatin, aprotinin, and leupeptin 1 μg ml^-1^) and centrifuged at 6500 RCF at 4°C for 15 min. Enzymatic reaction products were determined in duplicate by spectrophotometry at 405 nm, the assay sensitivity being 8 pg/ml. Results were expressed as ng/g dry weight tissue.

#### Plasmatic Nitrites Levels

Nitrates plus nitrites levels in plasma samples were determined according to Guevara’s method for the determination of nitrite/nitrate by Griess reaction (Guevara et al., 1998). 100 microliter of plasma were incubated with 10 μl of nitrate reductase (500 mU/ml) and 10 μl of NADPH (860 μM) for 2 h at 37°C, in the dark, to reduce nitrates to nitrites. After reduction, 240 μl of methanol/diethyl ether solution (3:1 v/v) were added to each sample and incubated overnight to induce protein denaturation and precipitation. The following day, samples were centrifuged for 10 min at 12500 RCF at 4°C, 100 μl of the supernatant were mixed with 25 μl of HCl 6N and 25 μl of 37.5 M sulphanilic acid and incubated for 10 min at room temperature. Finally, 25 μl of 12.5 M 1-naftilendiammine were added to each sample and incubated for 30 min at room temperature. The absorbance of the samples were measured in duplicate by spectrophotometry at 550 nm and expressed as μg/ml of plasma, the assay sensitivity being 100 ng/ml.

#### Colonic Caspase-3 and HSP70 Levels

Samples of colon were homogenized for 1 min in 1 ml of ice-cold PBS (pH 7.4) solution containing protease inhibitors cocktail (pepstatin, aprotinin, and leupeptin 1 μg ml^-1^) and centrifuged at 3000 RCF at 4°C for 15 min. The supernatant was collected, total protein concentration was quantified using Pierce BCA protein assay kit (ThermoFisher Scientific Inc., MA, USA) and an aliquot of the supernatant containing 100 mg proteins/ml (for Caspase-3 assay) and 200 mg proteins/ml (for HSP70 assay) were tested in commercially available ELISA kits (PRODOTTI GIANNI S.p.A., Milano, Italy), according to the manufacturer’s protocols. Each sample was assayed in duplicate and the optical density of the final enzymatic reaction products was determined at 450 nm. The sensitivity of the assays was 0.156 ng/ml (Caspase-3) and 200 pg/ml (HSP70). Results were expressed as ng/mg protein (Caspase-3) or as pg/mg protein (HSP70).

#### Intestinal Histology

Samples of colon harvested from normal animals and from colitic mice administered with saline, Ketanserin 5 mg/kg and 8-OH-DPAT 1 mg/kg were flushed with saline, immersion-fixed in 10% neutral buffered formalin overnight, dehydrated and embedded in paraffin. For each sample, at least five transverse 5-μm sections were cut both in the proximal and in the distal colon, stained with hematoxilin-eosin and examined in a light microscope (Nikon Eclipse E800) by a person unaware of the treatment. To assess histological injury in each section, the severity of mucosal destruction (0, normal; 1, mild; 2, moderate; 3, severe) and the extent of leukocytes infiltration in the lamina propria and submucosa (0, absent; 1, mild; 2, pronounced) were graded and the sum of scores was calculated (maximum score: 7) (modified from Bischoff et al., 2009). Since histological injury produced by TNBS instillation was localized almost exclusively in the distal portion of the colon, the average value of histological score was determined only from sections of distal colon, pooled with those determined for the other animals of the same experimental group and the mean value ± SEM was calculated.

### Statistical Analysis

All data were presented as means ± SEM. Comparison among groups were made using analysis of variance (one-way ANOVA) followed by Bonferroni’s post-test. Non-parametric Kruskal-Wallis analysis followed by Dunn’s post-test was applied for statistical comparison of DAI, MS, and cytokines levels. *P* < 0.05, *P* < 0.01, and *P <* 0.001 showed, respectively, statistically significant, highly significant, or extremely highly significant differences. All analyses were performed using Prism 4 software (GraphPad Software Inc. San Diego, CA, USA).

### Drugs

WAY100135 (5-HT_1A_ antagonist), GR125487 (5-HT_4_ anta gonist), and SB-269970 (5-HT_7_ antagonist) were purchased from Tocris Bioscence (Bristol, UK). Ondansetron (5-HT_3_ antagonist) was purchased from Glaxo Wellcome (Uxbridge, Middlesex, UK). 8-OH-DPAT (5-HT_1A_ agonist), Ketanserin (5-HT_2A_ antagonist), TNBS, MPO and all the other chemicals of reagent grade were purchased from Sigma–Aldrich Chemical Company (St. Louis, MO, USA). Drugs were dissolved in saline solution the day of the experiment.

## Results

### TNBS Induced Severe Colitis in Mice

**FIGURE 1:**
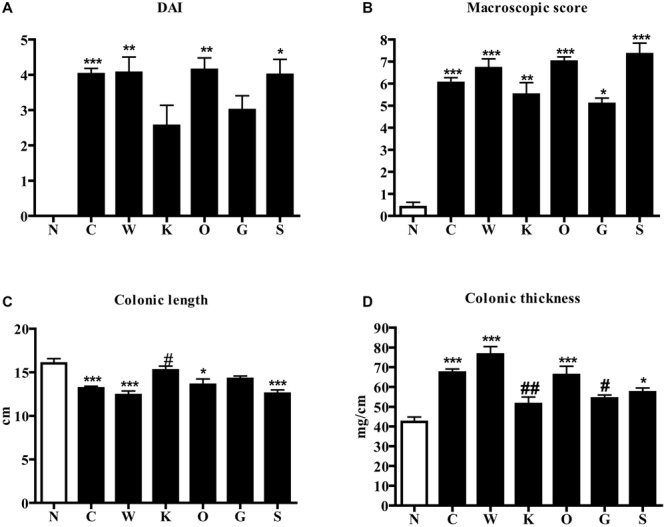
**Effects of 5-HT receptor antagonists on TNBS-induced disease severity.** DAI at day 4 **(A)**, MS **(B)**, colonic length **(C)**, and colonic thickness **(D)** assessed in normal mice (N) and in TNBS-treated mice administered with vehicle (C), WAY100135 5 mg/kg (W), Ketanserin 5 mg/kg (K), Ondansetron 10mg/kg (O), GR125487 10 mg/kg (G) and SB269970 10 mg/kg (S) (*n* = 6–12 data per group). ^∗^*P* < 0.05, ^∗∗^*P* < 0.01, ^∗∗∗^*P* < 0.001 vs. N mice; ^#^*P* < 0.05, ^##^*P* < 0.01 vs. C mice; one-way ANOVA followed by Bonferroni’s post-test. Kruskal–Wallis analysis followed by Dunn’s post-test was applied for statistical comparison of DAI and MS.

Intrarectal administration of the haptenating agent TNBS in saline-treated mice induced a global worsening of health conditions, expressed as DAI index, with respect to N animals: progressive reduction of body weight and loss of stool consistency were evident and reached their maximum at day 4, when animals were sacrificed (*P* < 0.001 Dunn’s test, **Figure 1A**). Macroscopic damage scores, based on the presence of adhesions, points of stenosis, mucus, erythemas, and ulcers in colon specimens were significantly increased after induction of TNBS colitis compared with N animals, that had only sporadically and scarcely visible rectal erythemas (*P* < 0.001 Dunn’s test, **Figure 1B**); colonic length was markedly reduced (*P* < 0.001 Bonferroni’s test, **Figure 1C**) and, conversely, thickness (*P* < 0.001 Bonferroni’s test, **Figure 1D**) and edema (5.3 ± 0.1 C vs. 3.6 ± 0.3 N group, *P* < 0.001 Bonferroni’s test) augmented following TNBS administration.

**FIGURE 2:**
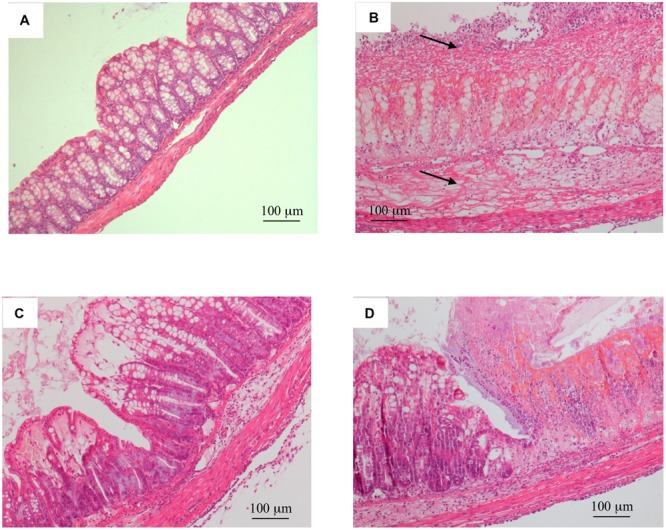
**Histology.** Representative hematoxylin-eosin stained sections of colonic specimens harvested from normal mice **(A)** and from TNBS-treated mice administered with vehicle **(B)**, Ketanserin 5 mg/kg **(C)** or 8-OH-DPAT 1 mg/kg **(D)**. TNBS colonic instillation caused epithelial degeneration, neutrophilic infiltration, and submucosal edema (indicated by arrows) in vehicle-treated animals **(B)**, not overtly modified either by Ketanserin **(C)** or 8-OH-DPAT **(D)** treatment.

Consistent with these alterations, the microscopic analysis revealed diffuse epithelial degeneration and massive neutrophilic infiltration of the distal colonic wall in TNBS-inoculated mice (**Figure 2B**; histological score: 6.0 ± 1.0) with respect to normal animals (**Figure 2A**; histological score: 0).

**FIGURE 3:**
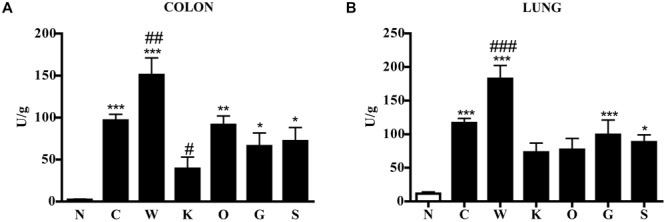
**Effects of 5-HT receptor antagonists on TNBS-induced neutrophil infiltration in colon and lung.** MPO activity in colonic **(A)** and lung **(B)** tissues excised from normal mice (N) and from TNBS-treated mice administered with vehicle (C), WAY100135 5mg/kg (W), Ketanserin 5mg/kg (K), Ondansetron 10mg/kg (O), GR125487 10mg/kg (G), and SB269970 10mg/kg (S) (*n* = 6–12 data per group). ^∗^*P* < 0.05, ^∗∗^*P* < 0.01, ^∗∗∗^*P* < 0.001 vs. N mice; ^#^*P* < 0.05, ^##^*P* < 0.01, ^###^*P* < 0.001 vs. C mice; one-way ANOVA followed by Bonferroni’s post-test.

**FIGURE 4:**
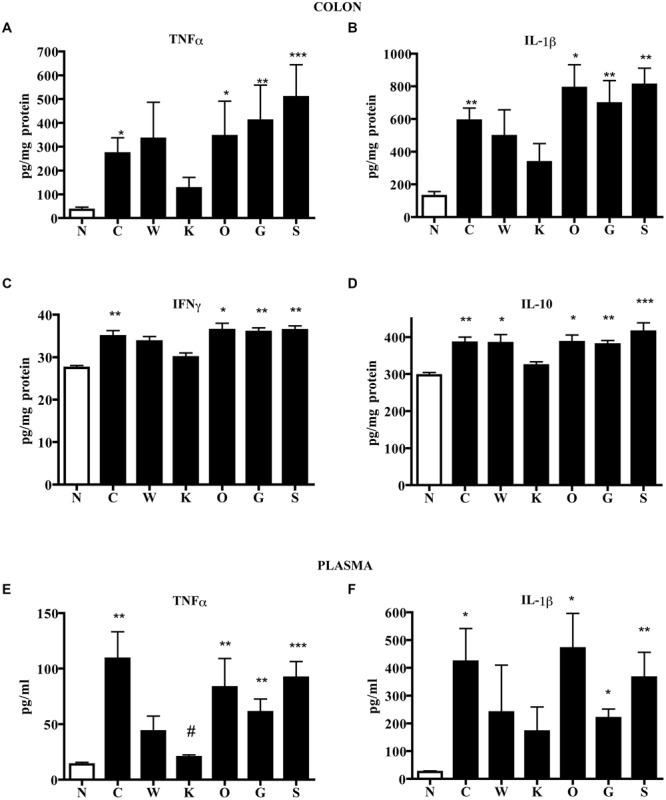
**Effects of 5-HT receptor antagonists on cytokines levels.** Colonic concentrations of TNFα **(A)**, IL-1β **(B)**, IFNγ **(C)**, and IL-10 **(D)** and plasmatic levels of TNFα **(E)** and IL-1β **(F)** in normal mice (N) and in TNBS-treated mice administered with vehicle (C), WAY100135 5 mg/kg (W), Ketanserin 5 mg/kg (K), Ondansetron 10 mg/kg (O), GR125487 10 mg/kg (G) and SB269970 10 mg/kg (S) (*n* = 6–12 data per group). ^∗^*P* < 0.05, ^∗∗^*P* < 0.01, ^∗∗∗^*P* < 0.001 vs. N mice; ^#^*P* < 0.05 vs. C mice; Kruskal–Wallis analysis followed by Dunn’s post-test.

These local morphological changes were accompanied by systemic inflammatory responses, represented by increased liver edema (2.20 ± 0.02 vs. 1.84 ± 0.02 N group, *P* < 0.001 Bonferroni’s test), intense infiltration of leukocytes in the colon as well as in lungs, witnessed by the increase in MPO activity (*P* < 0.001 vs. N Bonferroni’s test, **Figure 3**), and by the remarkable up-regulation of pro- and anti-inflammatory cytokines both in colonic tissues and in plasma of colitic mice (**Figure 4**). In this condition of severe colonic inflammation, tissue 5-HT content (*P* < 0.001 Bonferroni’s test) and plasmatic nitrites (*P* < 0.05 Bonferroni’s test) were more than doubled in C mice compared to N animals (**Table 1**).

**Table 1:** 5-HT, nitrites, and SP levels in plasma and colonic samples excised from normal mice (N) and from colitic mice administered with saline (C), WAY100135 5 mg/kg (W), and 8-OH-DPAT 1 mg/kg (OH) (*n* = 6–12 data per group).

	*N*	*C*	*W*	*OH*
Colonic 5-HT (g/g)	6.5 ± 0.8	16.1 ± 1.1^a^	14.7 ± 1.4^b^	11.2 ± 2.0^c^
Plasmatic nitrites (g/ml)	0.5 ± 0.1	1.1 ± 0.1^d^	1.6 ± 0.2^a,c^	1.3 ± 0.1^b^
Colonic SP (ng/g)	99.4 ± 13.5	260.9 ± 52.4	235.6 ± 41.9	215.1 ± 25.0

*Data are mean ± SEM. ^a^*P* < 0.001; ^b^*P* < 0.01 vs. N mice; ^c^*P* < 0.05 vs. C mice; ^d^*P* < 0.05 vs. N mice – one-way ANOVA followed by Bonferroni’s post-test.*

### 5-HT_2A_ Blocker Attenuated the Severity of TNBS-Induced Colitis

Among the different 5-HT receptor antagonists tested, only repeated administration of 5-HT_2A_ antagonist Ketanserin 5 mg/kg slightly improved health conditions of colitic mice and counteracted both colon shortening (*P* < 0.05) and thickening (*P* < 0.01) produced by TNBS intrarectal instillation, even if without reducing the MS (**Figure 1**) or the microscopic damage (**Figure 2C**; histological score: 6.3 ± 0.7).

Blockade of 5-HT_2A_ receptors significantly contrasted the infiltration of the TNBS-inflamed intestine with polymorphonuclear leukocytes (*P* < 0.05) and dampened the increase in lung MPO activity exhibited by colitic mice (**Figure 3**). The anti-inflammatory action of Ketanserin was evident also when considering the levels of bowel and plasmatic cytokines: indeed, the 5-HT_2A_ blocker was able to globally revert the increase in inflammatory cytokines evoked in TNBS colitis both in colon and in plasma (**Figure 4**), where TNFα and IL-1β concentrations were reduced, respectively, up to 5 (*P* < 0.05) and 2.5 times compared to C animals.

**FIGURE 5:**
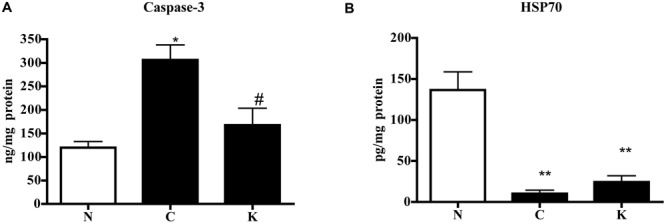
**Effect of Ketanserin on colonic caspase-3 and HSP70 levels.** Colonic concentrations of caspase-3 **(A)** and HSP70 **(B)** in normal mice (N) and in TNBS-treated mice administered with vehicle (C) or Ketanserin 5 mg/kg (K) (*n* = 6–12 data per group). ^∗^*P* < 0.05, ^∗∗^*P* < 0.01 vs. N mice; ^#^*P* < 0.05 vs. C mice; one-way ANOVA followed by Bonferroni’s post-test.

In order to investigate the possible mechanism underlying the protective effects displayed by Ketanserin, colonic levels of caspase-3, apoptotic marker, and of HSP70, a stress-inducible protein with cytoprotective properties, were determined in N, C, and Ketanserin-treated mice. As expected, TNBS enema produced a remarkable increase of caspase-3 levels and a highly significant reduction of HSP70: Ketanserin was able to counteract caspase-3 rise (*P* < 0.05 vs. C) but it did not affect HSP70 decrease (**Figure 5**).

### 5-HT_1A_ Blocker Worsened Neutrophil Infiltration in TNBS-Induced Colitis

Subcutaneous administration of 5-HT_1A_ antagonist WAY100135 10 mg/kg to TNBS-treated mice did not modify significantly the progression of the pathology nor the macroscopic damage score, slightly increasing only colon wall thickness in comparison with C mice (**Figure 1**). However, both intestinal and lung tissues excised from animals administered with 5-HT_1A_ blocker exhibited a conspicuous up-regulation of MPO activity, an index of neutrophil recruitment, with respect to vehicle-treated mice: in fact, treatment with WAY100135 nearly doubled MPO levels from 96.7 ± 7.3 U/g in the gut and 116.6 ± 6.9 U/g in lungs to, respectively, 150.8 ± 20.3 U/g and 182.6 ± 19.7 U/g (**Figure 3**). 5-HT_1A_ antagonist did not overtly modify the increased amount either of local or systemic inflammatory cytokines produced by TNBS (**Figure 4**).

### 5-HT_1A_ Agonist Weakly Mitigated the Severity of TNBS-Induced Colitis

**FIGURE 6:**
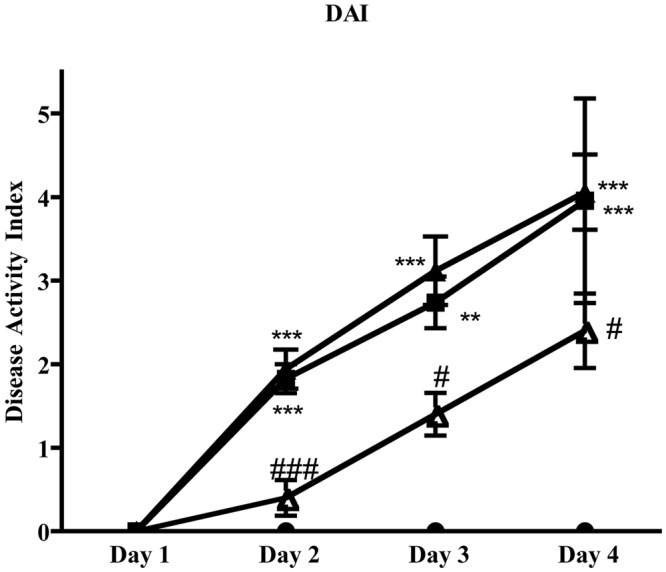
**Effects of 5-HT_1A_ ligands on disease progression.** Colitis severity, expressed as DAI, assessed on all four days following TNBS i.r. instillation in normal mice (black circles) and in mice administered with vehicle (black squares), WAY100135 5 mg/kg (black triangles) and 8-OH-DPAT 1mg/kg (white triangles) (*n* = 6–12 data per group). ^∗∗^*P* < 0.01, ^∗∗∗^*P* < 0.001 vs. N mice; ^#^*P* < 0.05, ^###^*P* < 0.001 vs. C mice; Kruskal–Wallis analysis followed by Dunn’s post-test.

**FIGURE 7:**
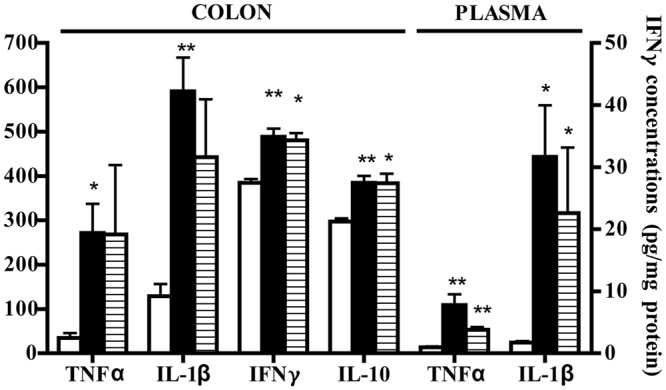
**Effects of 8-OH-DPAT on cytokines levels.** Colonic concentrations (pg/mg protein) of TNFα, IL-1β, IFNγ, and IL-10 and plasmatic levels (pg/ml) of TNFα and IL-1β in normal mice (white bars) and in TNBS-treated mice administered with vehicle (black bars) and 8-OH-DPAT 1mg/kg (gray bars) (*n* = 6–12 data per group). ^∗^*P* < 0.05, ^∗∗^*P* < 0.01 vs. N mice; Kruskal–Wallis analysis followed by Dunn’s post-test.

Administration of 5-HT_1A_ agonist 8-OH-DPAT 1 mg/kg to TNBS-treated mice remarkably improved their health conditions, DAI scoring being significantly lower than that of vehicle-treated mice every day of the observation period (**Figure 6**). Moreover, exogenous stimulation of 5-HT_1A_ receptors attenuated the thickening (55.0 ± 2.4 mg/cm vs 68.7 ± 3.4 C mice, *P* < 0.01) and the increase in colonic edema (4.8 ± 0.2 vs. 5.4 ± 0.1 C mice, *P* < 0.05) observed in segments excised from control mice, but did not reduce the mucosal damage, macroscopically or histo-pathologically detected (**Figure 2D**; histological score: 6.0 ± 1.0), nor the local and systemic leukocyte infiltration (data not shown) or cytokines release (**Figure 7**).

Interestingly, bowel 5-HT content was significantly decreased following 8-OH-DPAT treatment with respect to vehicle-treated colitic animals (*P* < 0.05, **Table 1**). Along with 5-HT, we investigated the role of 5-HT_1A_ receptors in affecting the release also of other mediators typically involved in intestinal inflammation. Accordingly, the levels of plasmatic nitrites and of colonic SP were assessed following 5-HT_1A_ stimulation and blockade in TNBS-treated mice: while 5-HT_1A_ antagonist significantly augmented the levels of nitrites when compared with N (*P* < 0.001) or C mice (*P* < 0.05), 8-OH-DPAT did not modify either nitrites or SP levels (**Table 1**).

### Blockade of 5-HT_3_, 5-HT_4_, and 5-HT_7_ Receptors Did Not Remarkably Affect the Severity of TNBS-Induced Colitis

The administration of 5-HT_4_ antagonist GR125487 10 mg/kg reduced DAI index at day 4 and colon thickness (*P* < 0.05) with respect to C mice (**Figure 1**), but had no clear effect on the other local and systemic inflammatory responses. Blockade of 5-HT_3_ receptors by Ondansetron 10 mg/kg and 5-HT_7_ receptors by SB269970 10 mg/kg provoked an increase in mortality rate of, respectively, 40 and 25% (data not shown), preventing experiments at higher dosages. Neither of the treatments applied were able to significantly aggravate or improve any of the clinical, macroscopic and biochemical parameters examined.

## Discussion

The results collected in the present study indicate that:

• TNBS intrarectal instillation in mice produced a strong local and systemic inflammatory response, associated with increased colonic levels of 5-HT;
• improved global health conditions, reduced morphological colonic changes and down-regulation of neutrophil recruitment and of inflammatory cytokines levels were produced by antagonism of 5-HT_2A_ receptors;
• blockade of 5-HT_1A_ receptors worsened neutrophil recruitment while, conversely, exogenous stimulation of the same receptor subtype delayed and mitigated the severity of TNBS-induced colitis, contrasting the increase in colonic 5-HT content;
• blockade of 5-HT_3_, 5-HT_4_, and 5-HT_7_ receptors did not remarkably affect the progression and outcome of the pathology or only slightly improved it.

The central role of 5-HT in modulating GI peristaltic and secretory reflexes has been known for a long time (Tonini, 2005); more recently, the influence of 5-HT also on innate and adaptive immune responses and on enteric inflammation has been emerging. The clinical and experimental studies currently available indicate a pro-inflammatory contribution of the monoamine to the pathogenesis of intestinal disorders like IBD (El-Salhy et al., 1997; Bischoff et al., 2009; Ghia et al., 2009; Margolis et al., 2014), although the mechanisms underlying and the receptor subtypes mediating this effect have yet to be identified.

In order to shed some light on the role played in intestinal inflammation by the 5-HT receptor subtypes most abundantly expressed in the GI tract, we chose a conventional chemical model of experimental colitis mainly driven by a Th1-mediated immune response, although well aware that no single model can perfectly mimic human IBD complexity (Kiesler et al., 2015). As expected, intrarectal TNBS challenge in mice produced a severe state of illness accompanied by colonic and systemic inflammatory responses and, consistent with the literature, by a higher 5-HT tissue amount (Oshima et al., 1999; Linden et al., 2003). Therefore, our findings apparently represent a new clue in favor of the pro-inflammatory role of the monoamine. However, when the overall results of the present investigation were examined, a more complex picture emerged, actually mirroring a multifaceted role of endogenous 5-HT in intestinal inflammation.

Repeated treatment of TNBS-challenged mice with different 5-HT receptor antagonists produced heterogeneous results, showing that endogenous 5-HT could provide simultaneously a deleterious and, alternatively, a beneficial contribution. Among the various 5-HT blockers applied, only 5-HT_2A_ antagonist Ketanserin protected animals against TNBS-induced colitis, attenuating the gravity of the local alterations and of the systemic inflammatory state. The ability of Ketanserin to markedly prevent both the colon shortening and the development of an inflammatory infiltrate, consisting presumably mainly of polymorphonuclear leukocytes, seems to indicate that, once released by EC cells following TNBS challenge (Bischoff et al., 2009; Margolis et al., 2014), endogenous 5-HT may participate in the up-regulation of colonic and plasmatic inflammatory cytokines and, consequently, to the recruitment of activated phagocytes in the bowel through the stimulation of 5-HT_2A_ receptors. 5-HT_2_ receptors have been described to influence several immune responses, by promoting the chemotaxis of immature dendritic cells (Muller et al., 2009) and eosinophils (Boehme et al., 2004) and activating CD4^+^ and CD8^+^ T lymphocytes (Inoue et al., 2011); conversely, blockade of the same receptor subtype by Ketanserin was able to counteract the up-regulation of inflammatory cytokines in a model of systemic inflammation (Sugino et al., 2009). Our present findings are in line with these observations and point toward the beneficial immunomodulatory effects of selective 5-HT_2A_ pharmacological blockade.

Besides the reduction in pro-inflammatory cytokines levels, the amelioration induced by Ketanserin on TNBS colitis may depend also on its anti-apoptotic effects on colonic tissues. Indeed, intestinal epithelial cells apoptosis is increased in the mucosa of IBD patients and in experimental colitis, although it is still unclear whether excessive cell death is the origin or simply a consequence of the inflammatory environment promoted in IBD (Günther et al., 2013); as a result, anti-apoptotic effects are often considered decisive to the protection afforded by a number of different therapeutic strategies in experimental colitis (see for instance Crespo et al., 2012; Arumugam et al., 2015; Yang et al., 2015). Interestingly, beneficial anti-apoptotic effects were demonstrated also by 5-HT_2_ antagonists in myocardial injury (Rajesh et al., 2006; Bharti et al., 2015). Altogether, our data lend support to a potential application of 5-HT_2A_ antagonists as supplemental strategy, combining anti-inflammatory and anti-apoptotic actions, for the treatment of intestinal inflammatory disorders.

Blockade of 5-HT_4_ receptors by GR125487 only partially overlapped the protective action of Ketanserin, weakly improving clinical conditions and attenuating colonic thickening: it is likely that the localization of 5-HT_4_ receptors on dendritic cells and their activating effects on immune cells functions (Idzko et al., 2004) could come into play in this regard.

If, in our experimental conditions, endogenous activation of 5-HT_2A_ and 5-HT_4_ receptors seems to contribute to the TNBS-induced damage, simultaneous stimulation of 5-HT_1A_ sites apparently contrasts the neutrophil recruitment triggered both locally and systemically by TNBS challenge, as demonstrated by the enhancing effect shown by 5-HT_1A_ antagonist WAY100135 on the strong leukocytes migration in colon and lungs induced by the instillation of the haptenating agent. Consistent with these findings is the protective action displayed by 5-HT_1A_ agonist 8-OH-DPAT, which improved the general health conditions of colitic mice and attenuated the wall structural alterations elicited by TNBS application. This advantageous effect exerted by stimulation of 5-HT_1A_ sites is not easily interpreted by taking into account their immunomodulatory actions documented *in vitro*, such as the inhibition of T cell proliferation and activity produced by 5-HT_1A_ antagonism (Aune et al., 1994). On the other hand, it is well known that 5-HT_1A_ receptors are present also on neural elements in the gut, where they pre-synaptically inhibit the release of Ach and of non-cholinergic neurotransmitters (LePard et al., 2004). Following this line of evidence, we tested the hypothesis that activation of 5-HT_1A_ receptors could interfere with the release of SP, a neuropeptide documented to be up-regulated and directly involved in IBD-induced intestinal inflammation (Gross and Pothoulakis, 2007). On the basis of the findings here collected we could rule out this possible mechanism of action, since neither 5-HT_1A_ agonist nor antagonist produced significant changes in the levels of colonic SP.

As regards the effects of 5-HT_1A_ receptor modulation on NO availability, several investigations documented a greatly increased production of this labile mediator in IBD and in experimental colitis and highlighted its pro-inflammatory and detrimental role or vice-versa its homeostatic and protective action in the pathogenesis of intestinal injury (Perner and Rask-Madsen, 1999; Kolios et al., 2004). In our conditions, the increased levels of plasmatic nitrites, stable metabolites of NO, detected in TNBS-colitis seem to support the potential harmful effects of this mediator; blockade of 5-HT_1A_ receptors further augmented the amount of NO, likely contributing to the negative outcome of WAY100135 treatment. On the whole, we can speculate that endogenous 5-HT may participate via 5-HT_1A_ receptors in limiting the massive neutrophils recruitment triggered in TNBS-colitis possibly by restraining NO over-production.

Interestingly, 5-HT_1A_ receptors have been localized also on EC cells (Kirchgessner et al., 1996) from which 5-HT can modulate its own release (Schworer and Ramadori, 1998). The efficacy exhibited by 8-OH-DPAT in reducing 5-HT colonic content could therefore reflect the role played by 5-HT_1A_ receptors in regulating 5-HT availability from EC cells in the inflamed gut. It is tempting to hypothesize that the beneficial effect provided by 5-HT_1A_ agonist in this model of TNBS-induced colitis might depend on the reduced intestinal 5-HT content, further highlighting the pro-inflammatory and deleterious role of the monoamine.

Finally and unexpectedly, neither blockade of 5-HT_3_ nor that of 5-HT_7_ receptors was able to significantly modify the disease progression and the intensity of the inflammatory response elicited by TNBS challenge. In fact, our results apparently contrast with recent investigations documenting the protection provided by 5-HT_3_ antagonists in TNBS-induced colitis in rats (Motavallian et al., 2013) and in 5-fluorouracil-induced intestinal mucositis in mice (Yasuda et al., 2013) and the controversial role, either protective or deleterious, described for 5-HT_7_ receptors in DSS-induced colitis (Kim et al., 2013; Guseva et al., 2014). However, differences between species, models of colitis and doses of SB-269970 administered could account for the observed discrepancies.

## Conclusion

Our findings shed new light on the double-faced role of 5-HT in intestinal delayed inflammation: the prevailing deleterious contribution mediated by 5-HT_2A_ and 5-HT_4_ receptors activation co-exists and is partially counteracted by 5-HT_1A_ stimulation, suggesting that agents targeting serotoninergic system could be beneficial as an additional therapeutic strategy in IBD only by carefully dissecting the monoamine antithetical effects.

## Author Contributions

AR carried out the studies and data analyses. SB performed the statistical analysis, interpreted the data, and wrote the manuscript. VA, FS, VV, and AG participated in performing the experiments and acquiring the data. AC carried out the histological analyses. EB conceived the study and supervised the investigation. All the authors revised the manuscript critically, approved its final version, and agreed to be accountable for all aspects of the work.

## Conflict of Interest Statement

The authors declare that the research was conducted in the absence of any commercial or financial relationships that could be construed as a potential conflict of interest.
